# Phosphorylation of the Archaeal Holliday Junction Resolvase Hjc Inhibits Its Catalytic Activity and Facilitates DNA Repair in *Sulfolobus islandicus* REY15A

**DOI:** 10.3389/fmicb.2019.01214

**Published:** 2019-05-31

**Authors:** Qihong Huang, Joseph Badys Mayaka, Qing Zhong, Chao Zhang, Guihua Hou, Jinfeng Ni, Yulong Shen

**Affiliations:** ^1^State Key Laboratory of Microbial Technology, Microbial Technology Institute, Shandong University, Qingdao, China; ^2^Cheeloo College of Medicine, Shandong University, Jinan, China

**Keywords:** archaea, protein phosphorylation, protein kinase, homologous recombination repair, holliday junction resolvase, Hjc

## Abstract

Protein phosphorylation is one of the main protein post-translational modifications and regulates DNA repair in eukaryotes. Archaeal genomes encode eukaryotic-like DNA repair proteins and protein kinases (ePKs), and several proteins involved in homologous recombination repair (HRR) including Hjc, a conserved Holliday junction (HJ) resolvase in Archaea, undergo phosphorylation, indicating that phosphorylation plays important roles in HRR. Herein, we performed phosphorylation analysis of Hjc by various ePKs from *Sulfolobus islandicus*. It was shown that SiRe_0171, SiRe_2030, and SiRe_2056, were able to phosphorylate Hjc *in vitro*. These ePKs phosphorylated Hjc at different Ser/Thr residues: SiRe_0171 on S34, SiRe_2030 on both S9 and T138, and SiRe_2056 on T138. The HJ cleavage activity of the phosphorylation-mimic mutants was analyzed and the results showed that the cleavage activity of S34E was completely lost and that of S9E had greatly reduced. *S. islandicus* strain expressing S34E in replacement of the wild type Hjc was resistant to higher doses of DNA damaging agents. Furthermore, SiRe_0171 deletion mutant exhibited higher sensitivity to DNA damaging agents, suggesting that Hjc phosphorylation by SiRe_0171 enhanced the DNA repair capability. Our results revealed that HJ resolvase is regulated by protein phosphorylation, reminiscent of the regulation of eukaryotic HJ resolvases GEN1 and Yen1.

## Introduction

DNA repair is the fundamental processes of life and is also interwined with other processes such as DNA replication, recombination, and CRISPR-Cas immunity in prokaryotes (Jones and Petermann, 2012; Faure et al., 2019). Proteins participating genetic information processing in archaea have served as structural models for understanding the mechanisms of DNA metabolism, including DNA replication, repair, recombination, and transcription in eukaryotes (Kelman and White, 2005; Werner, 2013). The studies for last two decades have shown that DNA repair pathways in archaea have both bacterial and eukaryotic-like proteins and, at the same time, they also have their own enzymes and features. For examples, early stage of HRR in archaea relies on Mre11 and Rad50 which are eukaryotic-like; while, further processing to generate 3′ssDNA overhang depends on the archaea-specific helicase/nuclease complex HerA-NurA (White, 2011). As another example, archaea lack the canonical bacterial and eukaryotic MutS-MutL MMR pathway, but they harbor a novel non-canonical protein EndoMS for mismatch recognition and repair (Ishino et al., 2016, 2018; Castaneda-Garcia et al., 2017).

Among several DNA repair pathways, HRR is most extensively studied and probably the most important pathway in archaea. Archaea contain homologs of eukaryotic HRR proteins and many HRR components (Mre11, Rad50, HerA, NurA, RadA, etc.) are essential for cell viability in thermophilic archaea, *Sulfolobus islandicus* and *Thermococcus kodakaraensis*, suggesting of an essential role of HRR in archaea (Fujikane et al., 2010; Huang et al., 2015b). Mre11-Rad50 initiates DNA end resection and degrades 5′ ssDNA in concert with HerA-NurA (Hopkins and Paull, 2008; Quaiser et al., 2008; Zhang et al., 2008; Blackwood et al., 2012; Rzechorzek et al., 2014). The resulting 3′ ssDNA is bound by RadA and strand invasion was performed with the help of several RadA paralogues, Rad54, RadC1 and RadC2 (Haseltine and Kowalczykowski, 2009; McRobbie et al., 2009; Liang et al., 2013; Wang et al., 2013). The SF-II helicase Hjm and PINA, a recently identified ATPase, are supposed to be responsible for HJ migration (Li et al., 2008; Woodman and Bolt, 2009; Song et al., 2016; Zhai et al., 2017). And HJ can be cleaved by a HJ resolvase (Komori et al., 1999). Besides HRR, the four way DNA intermediate HJ could be also generated via replication fork regression (Jones and Petermann, 2012; Krejci et al., 2012). HJ generated in stalled replication fork could be resolved by resolvases, generating DSB which is highly risky for the cell, or processed by replication fork reversal (Michel et al., 2018). The conserved HJ resolvase across all archaeal species is Hjc, while some *Sulfolobus* species contain an additional resolvase, Hje (Kvaratskhelia and White, 2000). Intriguingly, although Hje is not conserved, it has higher HJ DNA cleavage activity than Hjc and its deletion mutant exhibited higher sensitivity to DNA damaging agents in *S. islandicus* (Parker and White, 2005; Huang et al., 2015a). However, it is unclear how the cells regulate HJ resolution by different resolvases and in what situation that Hjc would work in archaea.

The complicated network of eukaryotic DNA repair pathways are frequently regulated by protein PTMs, especially protein phosphorylation which is involved in the DDR and DSBs repair at different levels (Sirbu and Cortez, 2013). DDR is mainly mediated by three key protein kinases, ATM (Ataxia telangiectasia mutated), ATR (ATM and Rad3-related), and DNA-PK (DNA-dependent protein kinase), all belonging to phosphoinositide 3-kinase (PI3K)-related kinases (PIKKs) family in eukaryotes (Blackford and Jackson, 2017). ATM is the apical kinase for global cellular responses to DSBs by phosphorylating hundreds of substrates involved in DNA repair ability, cell cycle checkpoint activation, apoptosis, chromatin remodeling, gene transcription, etc. (Shiloh and Ziv, 2013). A wide range of DNA lesions, especially ssDNA, can activate ATR for subsequent targeting its substrates that are shared with or unique to ATM (Ciccia and Elledge, 2010). ATM and ATR mediate DSBs repair via homologous recombination (HR), while DNA-PK is responsible for recruitment of NHEJ factors to DSBs for repairing (Jette and Lees-Miller, 2015).

A number of eukaryotic HRR proteins are phosphorylated by ATM or other kinases during repair, including each subunit of the MRN complex, Rad51, and HJ resolvases, etc. (Ciccia and Elledge, 2010). Interestingly, it has been shown that the HRR proteins Rad50, NurA, Hjm, and Hjc were phosphorylated in *S. acidocaldarius* (Reimann et al., 2013). In addition, all archaea encode ePKs, although the number is much smaller than that in eukaryotes (Kennelly, 2014). These promote us to ask whether HRR is also regulated by protein phosphorylation. In this study, we performed *in vitro* and *in vivo* study on the phosphorylation of Hjc by ePKs in *S. islandicus.* We demonstrate that Hjc was phosphorylated by three kinases at different sites *in vitro* and provide *in vivo* evidences that phosphorylation of Hjc by the Rio1 homolog, SiRe_0171, inhibits its catalytic activity and facilitates DNA repair in *Sulfolobus islandicus* REY15A.

## Materials and Methods

### Strains and Growth Conditions

*Sulfolobus islandicus* strain REY15A (E233S) (Δ*pyrEF*Δ*lacS*) (hereafter E233S) (Supplementary Table S1) and its transformants were cultured as described previously (Deng et al., 2009). D-arabinose [0.2% (wt/vol)] was used for induction of protein overexpression in *S. islandicus*. The chemical 5-fluoroorotic acid (5-FOA) was used for counter-selection of the *pyrEF* auxotroph.

### Plasmid Construction

#### Construction of Plasmids for Protein Overexpression in *Escherichia coli*

To construct the plasmids for expressing Hjc or its mutant proteins in *E. coli*, wide type *hjc* was amplified by PCR using the primers Hjc-*Nde*I-F/Hjc-*Sal*I-R (Supplementary Table S2). The Hjc mutant genes were constructed using splicing by overlap extension (SOE) PCR. The PCR product of each gene was digested with *Nde*I and *Sal*I and ligated into the *Nde*I and *Sal*I sites of the pET15bM vector [a modified version of pET15b (Shen et al., 2001)] to express Hjc protein without a tag.

#### Construction of Plasmids for Replacing Wild Type *hjc* With Hjc Mutant Genes in *S. islandicus*

The vectors for *in situ* expression of Hjc or its mutants in *S. islandicus* were constructed by amplification of each gene (or Hjc mutant genes) using Hjc-L-G-SOE-F/Hjc-*Mlu*I-R as the primers and their corresponding expression vectors as the templates. The gene was ligated with Hjc L-arm (amplified by Hjc-L-arm-*Sal*I-F/Hjc-L-G-SOE-R) by SOE PCR. The subsequent fragment was inserted into the *Sal*I and *Mlu*I sites of the vector pMID carrying the *pyrEF* marker (Peng et al., 2012), yielding pMID-Hjc-LG vector. The Hjc-R-arm was obtained by PCR using the primers Hjc-R-arm-*Nco*I/Hjc-R-arm-*Sph*I and inserted into the *Nco*I and *Sph*I sites of pMID-Hjc-LG after the restriction enzyme digestion and purification.

#### Construction of Plasmids for Gene Knockout by the CRISPR-Cas System

The plasmids for the knockout of kinase genes were constructed based on the vector pGE (from Prof. Qunxin She’s lab) (Li et al., 2016). Two complementary ssDNA of the protospacers (40 bp) within the target genes were synthesized by BGI (Beijing Genomics Institute, Beijing, China) and annealed. The resulting protospacer DNA was inserted into pGE between two repeat sequences, yielding pGE-Sp. The L-arm and R-arm for recombination to delete the target gene were amplified and joined by SOE PCR and the joined fragment was inserted into the *Sal*I and *Not*I sites of pGE-Sp after the restriction enzyme digestion and purification. The sequences of PCR primers are listed in Supplementary Table S2.

### Protein Purification

The pET15bM plasmids carrying *hjc* or the mutant genes were transformed into *E. coli* BL21 (DE3)-CodonPlus-RIL for protein expression. The procedure for protein induction and purification in *E. coli* cells was the same as previously described (Huang et al., 2017). Briefly, after induction, the cells were harvested and resuspended in buffer A (50 mM Tris–HCl pH 8.0, 200 mM NaCl, and 5% glycerol) for lysis by sonication. The soluble fractions were heated at 70^°^C for 30 min and after centrifugation the supernatants were purified by Hitrap^TM^ Heparin HP column and Superdex^TM^ 200 10/300 column sequentially (GE Health, United Kingdom). The protein concentration was determined by the Bradford method with bovine serum albumin (BSA) as the standard. Protein kinases were purified as described previously (Huang et al., 2017).

### *In vitro* Kinase Assay

For the phosphorylation activity assay of the protein kinases on Hjc (or its mutants), a certain mount (1 or 2 μM as specified) of wild type protein kinase and 5 μM Hjc was added into a reaction mixture (20 μl) containing 25 mM Tris–HCl pH 8.0, 10 mM NaCl, 5 mM MgCl_2_, 2 mM DTT, 50 μM ATP, and 8.3 nM [γ-^32^P]ATP (111 TBq/mmol, PerkinElmer). The mixture was incubated at 65^°^C for 30 min and the reaction was stopped by adding 5 × SDS-PAGE loading buffer and boiling for 10 min. The samples were analyzed by 12 or 15% SDS-PAGE as indicated. The autoradiographs were quantified by the software ImageQuant 5.2.

### DNA Substrates for the DNA Binding and Cleavage Activity Assays

Four oligonucleotides were synthesized for preparation of substrate for the HJ DNA binding and cleavage assays (Supplementary Table S2). Strand 5 (72-mer in length) was 5′ end labeled with γ-^32^P[ATP] and purified with Illustra^TM^ Microspin^TM^ G-25 column (GE Healthcare, United Kingdom) as previously described (Zhang et al., 2008). The HJ DNA substrate was constructed by combining Strand 5, 6, 7, and 8 (Supplementary Table S2) (Zhang et al., 2008). The DNA substrate was stored at 4^°^C.

### HJ DNA Binding Assay of Hjc

The HJ DNA binding assay was performed in 20 μl reaction mixtures consisting of indicated amounts of wild-type Hjc (or its mutants), 25 mM Tris–HCl pH 8.0, 25 mM NaCl, 5 mM MgCl_2_, 1 mM DTT, 10% glycerol, and 1 nM [γ-^32^P]-labeled HJ. The mixture was incubated at 37^°^C for 30 min followed by addition of a 5 × loading buffer (25% glycerol, and 0.025% bromophenol blue). The products were separated by electrophoresis in a 6% native polyacrylamide gel at 120 V for 90 min in 1 × TBE. The gels were exposed to a phosphorimager and scanned with Typhoon 9410.

### HJ DNA Cleavage Assay of Hjc

The HJ DNA cleavage assay was performed in 20 μl reaction mixtures consisting of indicated amounts of wild-type Hjc (or its mutants), 25 mM Tris–HCl pH 8.0, 25 mM NaCl, 5 mM MgCl_2_, 1 mM DTT, 0.01% BSA, and 1 nM [γ-^32^P]-labeled HJ. The mixture was incubated at 65^°^C for 30 min and then stopped by addition of a 2 × loading buffer (10 mM EDTA, 95% formamide, and 0.025% bromophenol blue). The products were boiled at 95^°^C for 10 min and analyzed on a 15% denatured polyacrylamide gel containing 7 M urea as previously described (Wei et al., 2008).

### Western Blot Analysis

Aliquots (50 μl for each) of the samples in the pull-down assay were mixed with 5 × SDS-PAGE loading buffer and loaded into a gel for SDS-PAGE analysis. The proteins in the PAGE gel were transferred onto a PVDF membrane at 30 mA for 16 hrs at 4^°^C. The membrane was washed and incubated with a primary antibody and then the secondary anti-rabbit HRP-conjugate antibody (HuaAn Biotechnology limited company, Hangzhou, China) following the standard protocol for Western blot. The band was visualized with Immobilon^TM^ Western Chemiluminescent HRP Substrate (Millipore Corporation, Billerica, MA, United States) and the image was obtained by Imagequant^TM^ 400 (GE Healthcare, United Kingdom).

### *In vitro* Pull-Down Assay

For pull-down assay, indicated amounts of Hjc (no His-tag) and SiRe_0171 (N-His) were mixed and incubated at 65^°^C for 30 min. The mixture was then mixed with 100 μl of Ni-NTA beads (Life Technologies, Carlsbad, CA, United States) pre-equilibrated with buffer A and incubated at RT for 10 min by gentle shaking. Unbound protein was removed by centrifugation at 3,000 *g* for 5 min. After being washed with 400 μl of wash buffer (buffer A supplemented with 40 mM imidazole) for four times, the His-tagged protein and its putative interacted protein were eluted with 200 μl of elute buffer (buffer A supplemented with 250 mM imidazole). The fractions were analyzed by SDS-PAGE and Western blot.

### Transformation and Gene Knockout of *S. islandicus*

The plasmids pMID for *in situ* expression of Hjc mutants were transformed into *S. islandicus* or Δ*hje* cells by electroporation as previously described (Deng et al., 2009). The transformants were selected in uracil-free medium and verified by PCR using the primers Hjc-Flanking-F/R (Supplementary Table S2). X-gal staining assay was performed as previously described (Huang et al., 2015b).

Kinase gene knockout was performed by transformation of the constructed pGE vectors into *S. islandicus* E233S for gene targeting by the CRISPR-Cas system. The gene deletion of subsequent culture was verified by PCR using the flanking primers (Supplementary Table S2). The culture was spread on the counter-selection plate to discard the pGE. The absence of the plasmids in the isolated colony was confirmed by PCR with plasmid-specific primers.

### DNA Damaging Agent Sensitivity Assay

To examine the sensitivity of the strains expressing different Hjc mutants or with kinase genes deleted to DNA damaging agents, cells were grown to early log-phase and transferred for 3–4 times before the spot assay. Cultures with initial OD_600_ value of 0.2–0.3 were diluted by 10-fold gradient dilutions for 5 times. An aliquot (10 μl) of each dilution was spotted on plates with or without treatment of 15, 25 J/m^2^ UV, or on plates containing methyl methanesulfonate (MMS, 3 and 4 mM), hydroxyurea (HU, 2 and 3 mM), or cisplatin (10 and 15 μg/ml). The plates were incubated at 75^°^C for 7–10 days. At least three independent experiments were performed and the representative figures were shown.

## Results

### Phosphorylation of Hjc by Different Protein Kinases From *S. islandicus*

**FIGURE 1:**
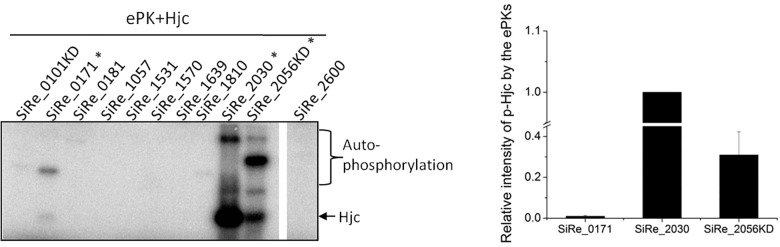
Hjc is phosphorylated by three eukaryotic-like protein kinases (ePKs) SiRe_0171, SiRe_2030, and SiRe_2056KD from *S. islandicus in vitro*. Each ePK (2 μM) was incubated with 5 μM Hjc in the reaction mixture containing [γ -^32^P]ATP at 65^°^C for 30 min (see the Materials and Methods). After reaction, the samples were analyzed by 15% SDS-PAGE. The experiments were performed for at least three times for each enzyme. Left panel, a representative image (15% gel) showing the activities of the ePKs autophosphorylation and phosphorylation on Hjc. The three ePKs that are able to phosphorylate Hjc are labeled with asterisks. Right panel, quantification of the phosphorylation activity of SiRe_0171, SiRe_2030, and SiRe_2056KD on Hjc. The vertical axis shows relative signal strength, the ratio of phosphorylated Hjc by each ePK divided by that by SiRe_2030. Standard deviation is indicated with error bars.

Previously, it was reported that the HJ resolvase Hjc, but not Hje, and several other DSBs repair proteins Rad50, NurA, and Hjm were phosphorylated in a phosphatase deletion mutant of *S. acidocaldarius* (Reimann et al., 2013). In order to understand the function and mechanism of the phosphorylation in archaea, we chose Hjc for *in vitro* and *in vivo* analysis. Previously, eleven ePKs were purified from *E. coli* or *S. islandicus*, and their autophosphorylation and cross-phosphorylation activities were characterized (Huang et al., 2017). To determine which ePK(s) was responsible for Hjc phosphorylation, *in vitro* phosphorylation activity assay using purified Hjc protein as the substrate was carried out to screen the kinases that were able to phosphorylate Hjc. Under our experimental conditions, at least three were able to phosphorylate Hjc (Figure 1), albeit with different activities. SiRe_2030 and SiRe_2056KD (kinase domain of SiRe_2056) exhibited higher activity than SiRe_0171 (2.23 ± 0.54 mmol ATP min^-1^ mol^-1^), which is about 1/100 and 1/30 of those of SiRe_2030 (209.62 ± 40.20 mmol ATP min^-1^ mol^-1^) and SiRe_2056KD (66.01 ± 18.30 mmol ATP min^-1^ mol^-1^), respectively (Figure 1). Interestingly, SiRe_0171 is a Rio kinase (LaRonde, 2014). Because SiRe_2030 and SiRe_2056KD seem to have broad substrate activity (Huang et al., 2017), we focused on SiRe_0171. To confirm that Hjc was indeed phosphorylated by SiRe_0171, a catalytic dead mutant, SiRe_0171D188N, was applied for the kinase assay. The result showed that the signal was only obtained in the presence of Hjc and wild type SiRe_0171 but not the mutant SiRe_0171D188N (Supplementary Figure S1A), and the phosphorylation was completed within 15 min for the wild type SiRe_0171 (Supplementary Figure S1B). To understand whether SiRe_0171 has physical interaction with Hjc, *in vitro* pull-down assay was performed. Hjc signal was detectable in the presence of N-His-SiRe_0171 by Western blot (Supplementary Figure S1C). The signal was not strong, probably due to the transient reaction of phosphorylation. The result indicates that SiRe_0171 has weak physical interaction with Hjc.

### Hjc Was Phosphorylated by Different ePKs at Various Residues

**FIGURE 2:**
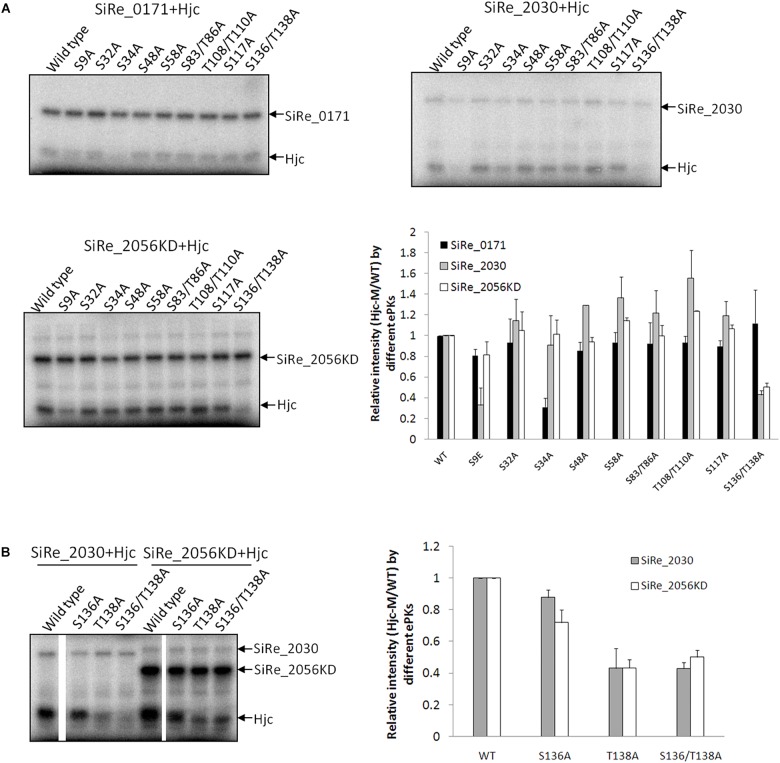
ePKs phosphorylate Hjc at different Ser/Thr residues. **(A)** Phosphorylation of various Hjc mutants by SiRe_0171, SiRe_2030, and SiRe_2056KD. Representative images of Hjc phosphorylation are shown. Quantification of the autoradiographs is shown at the right bottom. **(B)** Phosphorylation of Hjc mutants, S136A, T138A, and S136/T138A by SiRe_2030 and SiRe_2056KD. The data was obtained from three independent experiments. Error bars indicate standard deviation.

To determine whether the three ePKs phosphorylate Hjc at the same or different residues, we searched for all Ser or Thr residues within the amino acid sequence of Hjc and analyzed their conservation among the homologs. Hjc homologs from multiple archaeal species, especially those in the two mainly studied phyla, Crenarchaeota and Euryarchaeota, were aligned. There are eight Ser residues and four Thr residues with in *S. islandicus* Hjc (Supplementary Figure S2), among which two residues, S32 (19/19) and T108 (15/19), are conserved (Supplementary Figure S2). Firstly, each of the two conserved residues was mutated to Ala and the phosphorylation of these Hjc mutants by the three ePKs was examined. During the purification processes, the wild type Hjc and the two mutants (and others, see below) were all stable (no observation of precipitation) and eluted at the same volume in gel filtration, indicating that the Hjc mutants have same protein folding. However, their phosphorylation signal strengths by different ePKs were all comparable with that of the wild type Hjc. It seems that none of these two sites was the target of the ePKs. Next, we mutated the remaining Ser or Thr residues to Ala and generated various Hjc mutants with single or double mutations (Two Ser/Thr residues were mutated at the same time if they are close to each other), including S9A, S34A, S48A, S58A, S83/T86A, T108/T110A, S117A, S136/T138A. As shown in Figure 2A, phosphorylation of S34A by SiRe_0171 was reduced specifically, suggesting that S34 is the main target residue for SiRe_0171 (Figure 2A). This was confirmed by the reduced levels of phosphorylation of several double- or triple-point mutants containing S34A. The signal strength of any mutant containing S34A was significantly reduced, similar to that of the single point mutant S34A (Supplementary Figure S3).

Phosphorylation of all the nine Hjc mutants by SiRe_2030 and SiRe_2056KD was also examined. We found that S9 was one of the targets for SiRe_2030 (Figure 2A). In addition, the phosphorylation of S136A/T138A also decreased apparently in the presence of SiRe_2030 or SiRe_2056KD (Figure 2A,B). Further individual single point mutagenesis analysis revealed that both ePKs mainly phosphorylated T138 of Hjc (Figure 2B). SiRe_2056KD was also able to phosphorylate S136 to a less extent compared to T138. Thus, different *Sulfolobus* ePKs can phosphorylate Hjc at various residues: SiRe_0171 on S34, SiRe_2030 on both S9 and T138, and SiRe_2056KD mainly on T138. This is strikingly because eukaryotic protein kinases can also target one protein at various sites.

### Two Hjc Phosphorylation-Mimic Mutants Exhibited No or Reduced HJ DNA Cleavage Activity

**FIGURE 3:**
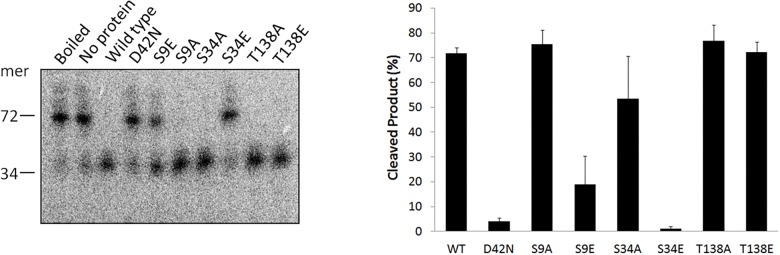
A phosphorylation-mimic Hjc mutant S34E completely lost the HJ DNA cleavage activity. Ten nanomolar wild type Hjc or its mutant was incubated with HJ at 65^°^C in 20 μl reactions (see the Materials and Methods). The samples were mixed with loading buffer, boiled for 10 min, and analyzed on a 15% denatured PAGE gel containing 7 M urea. The position of a 34-nt oligonucleotide (Supplementary Table S2) is indicated at the left side of the gel. The experiments were performed at least for three times. A representative image is shown as the left panel and the quantitation is shown as the right panel. Error bars indicate standard deviation.

In eukaryotes, phosphorylation of a substrate can have different effect on the target protein, such as affecting the protein conformation and substrate binding ability, and regulating the catalytic activity (Humphrey et al., 2015). Hjc is a conserved archaeal HJ resolvase exhibiting both HJ DNA binding and cleavage activities (Kvaratskhelia and White, 2000). To determine the effect of phosphorylation on Hjc, three phosphorylation-mimic mutants, S9E, S34E, and T138E, were constructed and purified. The *in vitro* activity assay for the non-phosphorylated mutants, S9A, S34A, and T138A, was also examined. The results showed all Hjc mutants still exhibited efficient HJ DNA binding capability, although they had slight difference from that of the wild type protein (Supplementary Figure S4). In contrast, only partial HJ DNA cleavage activity (about 1/3) was maintained by S9E and the cleavage activity of S34E was completely abolished, while the HJ DNA cleavage activity of phosphorylation-mimic mutants T138E was comparable with that of the wild type (Figure 3). Since we found that SiRe_2056KD had phosphorylation activity toward S136 besides T138 (Figure 2B), an Hjc mutant S136E/T138E was constructed and the *in vitro* activity assay showed that its cleavage activity decreased to a level similar with that for S9E (data not shown). On the other hand, non-phosphorylated mutants S9A, S34A, and T138A still exhibited efficient HJ DNA cleavage activity (Figure 3). To confirm that the effect of the three Glu mutants on the HJ DNA cleavage activity was due to Hjc phosphorylation, the cleavage activity of the wild type Hjc in the presence of each ePK was assayed and the results showed that the activity was indeed inhibited (Supplementary Figure S5). In conclusion, phosphorylation of Hjc had negative effect on the its nuclease activity of Hjc, but not on its HJ DNA binding capability.

**FIGURE 4:**
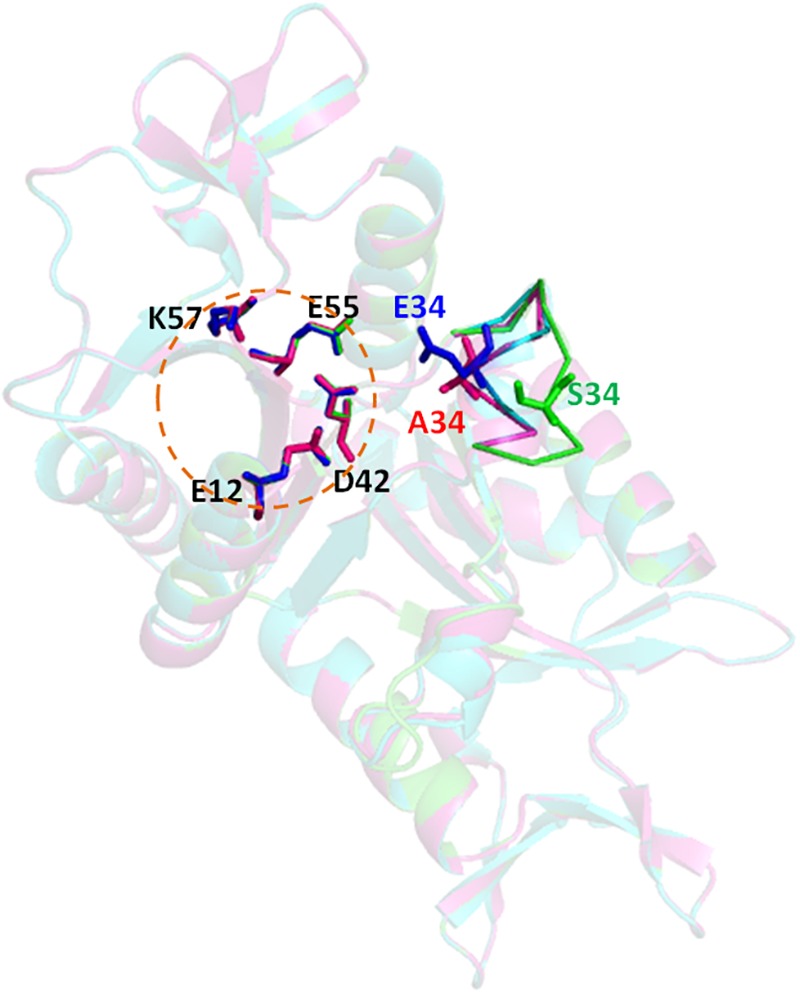
Structure modeling of Hjc, S34A, and S34E. The structural model were built using the online server SWISS-MODEL (https://swissmodel.expasy.org/) based on the *S. solfataricus* Hjc structure (PDB: 1HH1) and analyzed by PyMOL. The three structures (dimer) are aligned together in which wild type (S34), S34A, and S34E are in blue, pink, and green colors, respectively. The circular dotted line indicates the catalytic center consisting of four highly conserved residues, E12, D42, E55, and K57. A five-amino acid loop containing the mutated residue is shown as sticks. The distances between the 34th amino acid to the closest catalytic residue D42 are D42-S34∼9.9Å, D42-A34∼5.4Å, and D42-E34∼4.7Å, respectively.

To get an insight to the mechanism of phosphorylation effect on the cleavage activity of Hjc mutant S34E, structure homology-modeling was performed for the wild type Hjc and two mutants, S34A and S34E, based on the crystal structure of *S. solfataricus* Hjc (PDB: 1HH1). The catalytic center of Hjc consists of four highly conserved residues, E12, D42, E55, and K57 (Figure 4). A loop containing S34 in the wild type protein was ca. 9.9Å far from the closest catalytic residue D42. However, both A34 and E34 were close to D42 in which E34 was a bit closer: A34-D42∼5.4Å and E34-D42∼4.7Å in the mutants S34A and S34E, respectively (Figure 4). Ala is an amino acid without any side strand, whereas Glu contains a long negative charged side strand, similar to PO_4_^3-^, which would probably interfere with the catalytic center. The interference might disrupt Mg^2+^ binding and consequently inhibit Hjc cleavage activity.

### Hjc Phosphorylation-Mimic Mutant S34E Strain Is More Resistant to Higher Doses of Cisplatin and UV Treatment

Our *in vitro* studies revealed that Hjc phosphorylation at S34 inhibited its HJ cleavage activity, but it is still unclear what the *in vivo* role of the phosphorylation. In order to explore the physiological function of Hjc phosphorylation, strains expressing different Hjc mutants were constructed and their sensitivity to DNA damaging agents was analyzed. In these strains, the wild type *hjc* gene was replaced with the gene of S9A, S9E, S34A, S34E, T138A, or T138E using the strategy of marker replacement (Supplementary Figure S6).

**FIGURE 5:**
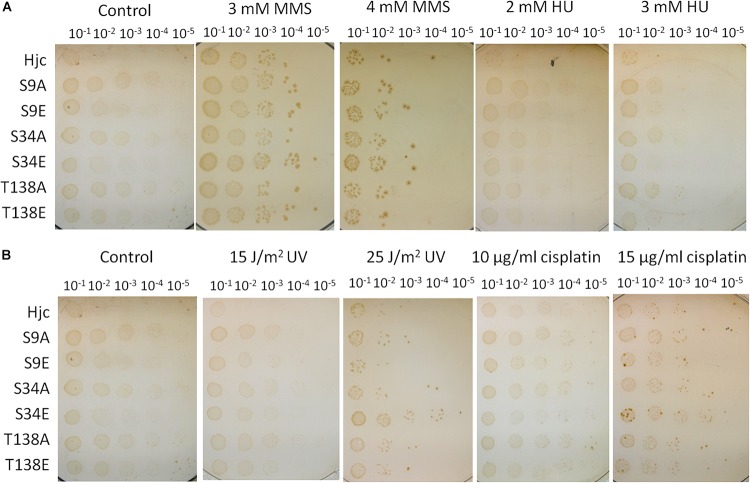
Assay of the sensitivity of the strains to DNA damaging agents. The strains were cultivated until OD_600_∼0.2 and serially diluted by 10 times. An aliquot of 10 μl for each diluted culture was spotted on the plates and treated with UV (15 and 25 J/m^2^, in **B**), or on plates containing methyl methanesulfonate (MMS, 3 and 4 mM, in **A**), hydroxyurea (HU, 2 and 3 mM, in **A**) or cisplatin (10 and 15 μg/ml, in **B**). The plates were incubated at 75°C for 7–10 days. The experiments were performed for 2–3 times. Representative figures are shown.

The sensitivity to DNA damaging agents of the wild type and mutant strains was analyzed by spot assay using different doses of MMS, HU, cisplatin, and UV. As shown in Figure 5, in the presence of MMS and HU, there were no apparent difference between the wild type strain and those expressing various Hjc mutants (Figure 5A). Surprisingly, although the cells grew normally in the presence of low doses of cisplatin (10 μg/ml) or UV (15 J/m^2^), the strain expressing S34E grew faster than other strains in the presence of relative high doses of cisplatin (15 μg/ml) or UV (25 J/m^2^), indicative of a higher DNA repair capability (Figure 5B).

We found that the strain expressing S9E or T138E exhibited comparable DNA repair capacity with the wild type strain (Figure 5). According to our previous genetic work result, Δ*hje*, but not Δ*hjc*, was sensitive to DNA damaging agents, indicative of a dominant role for Hje in DNA repair in the cell (Huang et al., 2015a). The presence of Hje may blanket the effect of Hjc mutants *in vivo*. To further analysis the effect of Hjc mutants, especially S9A/E and T138A/E, on the cell, the Δ*hje* strains expressing various Hjc mutants (Δ*hje*Δ*hjc:hjc-M*) were constructed using the same strategy of marker replacement. The Hjc mutant genes of the transformants were amplified by PCR for sequencing. The results showed that five Hjc mutant genes were originally designed except for S34E, which has no catalytic activity (Figure 3). The experiment was performed twice and the same result was obtained. This is consistent with our previous finding that *hje* and *hjc* could not be deleted simultaneously (Huang et al., 2015a), suggesting that at least one active HJ resolvase should be maintained for cell viability. DNA damaging agent sensitivity assay revealed that all of the five strains together with Δ*hje*Δ*hjc:hjc* exhibited higher sensitivity to the same dose of DNA damaging agents than the strains Δ*hjc:hjc-M* (Supplementary Figure S7), in agreement with our previous result that Δ*hje* was sensitive to DNA damaging agents. However, there is no apparent difference between Δ*hje*Δ*hjc:hjc* and other Δ*hje* strains expressing Hjc mutants (Supplementary Figure S7).

### The Deletion Strain of the Gene of ePK SiRe_0171 Exhibited Higher Sensitivity to High Doses of DNA Damaging Agents

**FIGURE 6:**
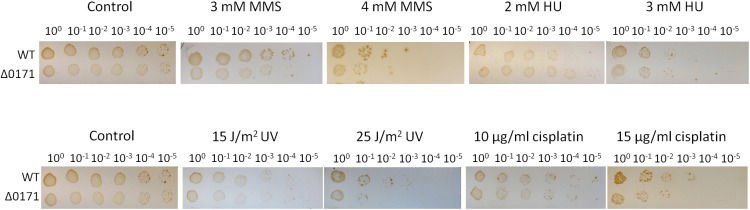
SiRe_0171 deletion mutants are more sensitive to higher doses of UV and cisplatin. The method for DNA damaging agent sensitivity assay was the same as that in Figure 4. The experiments were performed for three times. Representative figures are shown.

To confirm whether the enhanced resistance to DNA damage of the strain HjcS34E was the effect of SiRe_0171 phosphorylation, a mutant strain with SiRe_0171 deletion was obtained by the recently developed CRISPR-Cas genome-editing system (Supplementary Figure S8) (Li et al., 2016). The results of DNA damaging agent sensitivity assay showed that Δ*SiRe_0171* were more sensitive to relative high doses of cisplatin (15 μg/ml) or UV (25 J/m^2^), but not to HU or low doses of cisplatin (10 μg/ml) or UV (15 J/m^2^), than the wild type (Figure 6). This suggested that, without SiRe_0171 phosphorylation, the cells were difficult to deal with a larger amount of DNA lesions, in agreement with the result that S34E was resistant to high doses of DNA damaging agents. In addition, Δ*SiRe_0171* also exhibited sensitivity to MMS (Figure 6), indicative of a broader regulatory roles for SiRe_0171. The sensitivities of Δ*SiRe_0171* to cisplatin and MMS were also performed in the liquid medium and the results were similar to those from the plates containing the corresponding DNA damaging agents (Supplementary Figure S9).

## Discussion

Despite of over two decades of study, our knowledge of archaeal DNA repair is still limited in general. The networks of the DNA repair pathways are still incomplete for the investigated culturable archaea so far. Additionally, the regulation of DNA repair processes by PTM has not been even scratched until this study, and the DDR network has just begun to emerge (Sun et al., 2018). Recently, many novel archaea lineages have been discovered by metagenomic analysis, and the most outstanding one is the Asgard superphylum. Interestingly, accumulating evidences support a debated hypothesis that eukaryotes originated within archaea, in particular the Asgard superphylum (Zaremba-Niedzwiedzka et al., 2017). *Sulfolobus* are arguably the best studied model archaea in the investigation on the mechanisms of DNA metabolism, cell cycle, CRISPR-Cas immunity, and virus-host interaction (Kelman and White, 2005; Bernander, 2007; Snyder et al., 2013; Zhang and White, 2013). As a genus of Crenarchaeota, *Sulfolobus* belongs to TACK superphylum, which is close to Asgard superphylum. Therefore, the study of the model archaea *Sulfolobus* will certainly shade light into the biology of Asgard and many other unculturable archaea.

Very recently, an Orc1-2-centered DDR network has been uncovered in *Sulfolobus* (Sun et al., 2018). In the presence of DSBs, the Orc1-2 protein (an AAA + ATPase) is highly induced. It recognizes and binds to the promoter regions of DDR and Tfb3 genes and activates or represses their expression (Sun et al., 2018). Tfb3 upregulates a subset of gene expression including *mre11-herA-nurA-rad50* operon, *ups*, and *ced* gene transfer system, while represses those in DNA replication initiation, genome segregation, and cell division (Feng et al., 2018; Schult et al., 2018). However, many details of the network await investigation. In addition, it is unknown how the DNA response processes are regulated by PTM, particularly phosphorylation, since it was reported that a number of DNA repair proteins were phosphorylated *in vivo* (Reimann et al., 2013).

Here, for the first time, we showed that *S. islandicus* HJ resolvase Hjc was phosphorylated by three ePKs, SiRe_0171, SiRe_2030, and SiRe_2056KD. The ePKs targeted on different residues of Hjc. Phosphorylation of S34 by SiRe_0171 completely inhibited its cleavage activity. The strain expressing phosphorylation-mimic mutant HjcS34E resulted in enhanced cell growth in the presence of higher doses DNA damaging agents, whereas deletion of *SiRe_0171* repressed the growth under the same treatment. Our study has elucidated a regulation of an important enzyme Hjc, the nuclease dealing with DNA replication/recombination/repair intermediate.

We found that Hjc was phosphorylated mainly on three residues: SiRe_0171 on S34, SiRe_2030 on S9/T138, and SiRe_2056KD on T138, but none of the three phosphorylated residues are conserved in archaeal Hjc proteins according to the sequence alignment analysis (Supplementary Figure S2). A previous phospho-proteomic analysis in *S. acidocaldorius* showed that S58 is phosphorylated after a protein phosphatase (pp2a) was deleted (Reimann et al., 2013). However, S58 and two other conserved residues, S32 and T108, are not the target of the three ePKs above according to our *in vitro* kinase assay. In addition, we found that Hjc mutant S58A and S58E exhibited efficient HJ binding and cleavage activity similar to that for the wild type protein (data not shown). Therefore, it is still unclear whether S58 is phosphorylated in *S. islandicus* and, if so, what function for its phosphorylation. Although SiRe_2030 and SiRe_2056KD had high phosphorylation activity on Hjc, the two Hjc phosphorylation-mimic mutants S9E and T138E had slight or no effect on either its HJ cleavage activity or the binding activity. Furthermore, the strain expressing S9E or T138E, either in the presence or absence of Hje, exhibited comparable DNA repair capacity with the wild type strain. So, it may be needed to analyze the phenotype of a strain expressing Hjc containing double mutations (S9/T138E or S136/T138E). On the other hand, Hje phosphorylation was not reported in the phospho-proteomic analysis in *S. acidocaldorius* and our *in vitro* kinase assay revealed that SiRe_2030 and SiRe_2056KD, but not SiRe_0171, were also able to phosphorylate Hje (data not shown). So, we could not exclude that these two ePKs may target substrates through an unspecific interaction *in vitro*.

We revealed that SiRe_0171 (Rio1) deletion mutants were more sensitive to DNA damaging agents than the wild type strain. SiRe_0171 belongs to the atypical protein kinase family, Rio kinase, which is found in all three domains of life. In eukaryotes, RIO kinases participate in ribosome biogenesis, cell cycle progression, and genome integrity (Angermayr et al., 2002; Ferreira-Cerca et al., 2012; Widmann et al., 2012). Very recently, it was shown that the human RIO1 promoted tumor growth and was suggested as a potential therapeutic target since it was overexpressed in different tumor entities (Weinberg et al., 2017). Although it was suggested that archaeal Rio might have a conserved role in ribosome biogenesis, their function had not been established so far (Knuppel et al., 2018). However, several transcriptional reports showed that *S. solfataricus* Rio1 was induced at early stage of UV-treatment and the mRNA level of Rio1 increased in γ–irradiation-treated *Pyrococcus furiosus* cells (Gotz et al., 2007; Williams et al., 2007). These results are consistent with our data that SiRe_0171 is involved in DDR or DNA repair, in which phosphorylation of Hjc may be one of acting modes.

Eukaryotes contain several HJ resolvases and its HJ resolution is regulated by phosphorylation. *S. pombe* Mus81-Eme1 is activated for HJ resolution by Cdc2(CDK1)-primed, Rad3(ATR)-dependent phosphorylation in cells lacking BLM-related helicases (Dehe et al., 2013). The DNA binding activity of *S. cerevisiae* Yen1 is inhibited via phosphorylation of its nuclear localization signal (NSL) by Cdk in S phase and the protein is excluded from the nucleus. Dephosphorylation of Yen1 by Cdc14 at anaphase activates its binding activity and leads to nuclear re-localization for HJ processing (Blanco et al., 2014). However, the ortholog of Yen1 in human, GEN1, is regulated independent of phosphorylation. GEN1 is controlled by nuclear exclusion, driven by a nuclear export signal (NES) peptide that allows GEN1 to act only in mitosis when the nuclear membrane breaks down (Chan and West, 2014). These implied that regulation of HJ resolvases might originate early in evolution history of life, but the regulatory mechanisms were diversified during evolution.

**FIGURE 7:**
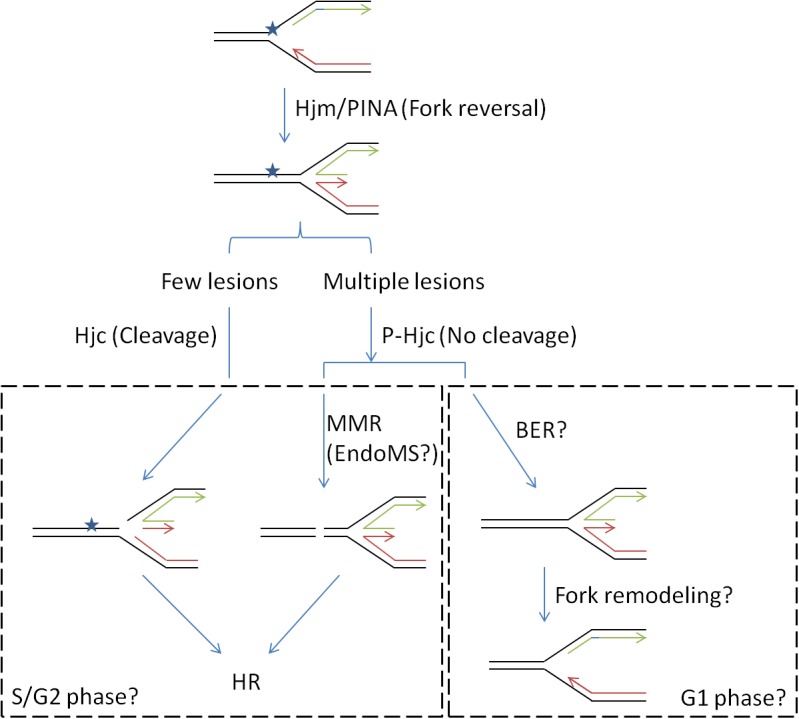
A proposed model for the function of Hjc-S34 phosphorylation in DNA repair (stalled replication fork). DNA replication fork is usually blocked by a lesion. The stalled replication fork would be reversed to a HJ DNA by proteins such as Hjm and/or PINA (PIN domain-containing ATPase), followed by the recruitment of Hjc (Zhai et al., 2018). Phosphorylation states of Hjc might control different repair pathways: Non-phosphorylated Hjc would cleave the HJ forming a DSB which would be repaired by HR; phosphorylated Hjc at S34 would not cleave the HJ and the lesion might be repaired by mismatch repair (MMR) or base excision repair (BER). The latter may occur in the presence of high amounts of DNA lesion when treated with higher dose of DNA damaging agents. It is also possible that the choice for different pathways depended on cell cycles where S/G2 phases contain two copies of genomes for HR. MMR is probably mediated by a newly identified nuclease EndoMS, resulting in formation of a DSB repaired by HR. The replication fork might be remodeled after the lesion was removed by BER.

In the current work, we demonstrated that expression of Hjc phosphorylation-mimic mutant S34E, which abolished its cleavage activity, stimulated cell growth in the presence of relatively high-dose, but not low-dose, UV or cisplatin. The DNA damaging agents induce inter- or intra-DNA crosslink which would impair DNA replication (Deans and West, 2011). The damages at the stalled replication forks may be repaired by translesion DNA synthesis, BER, MMR, or HR in different phases of cell cycle (Jones and Petermann, 2012). We have recently proposed a role of Hjc in repair of stalled replication fork in concert with Hjm and PINA (Zhai et al., 2018). The inhibition of Hjc activity by phosphorylation at S34 might avoid HJ cleavage and stalled replication fork collapse. This would allow the cell to repair the lesions rapidly in the presence of large amounts of DNA damages (Figure 7). It will be very interesting to find out whether Hjc phosphorylation and the expression of certain ePK are cell cycle-dependent, and whether the phosphorylation of Hjc by different ePKs mediates the interaction of Hjc with various partners in the cells (such as Hjm and PINA) for HJ processing in different stages of cell cycle, since SiRe_2030 and SiRe_2056 whose phosphorylation did not have apparent effect on Hjc activity.

## Data Availability

The raw data supporting the conclusions of this manuscript will be made available by the authors, without undue reservation, to any qualified researcher.

## Author Contributions

QH designed the project, conducted most of the experiments, analyzed the data, and wrote draft of the manuscript. JM and QZ performed part of the experiments in plasmid construction and protein purification. JM and JN helped revise the manuscript. CZ and GH provided the platform and technical support for γ-^32^P[ATP] manipulation. YS conceived the idea for the project and helped write the manuscript. All authors approved the version to be published and agreed to be accountable for all aspects of the work in ensuring that questions related to the accuracy or integrity of any part of the work are appropriately investigated and resolved.

## Conflict of Interest Statement

The authors declare that the research was conducted in the absence of any commercial or financial relationships that could be construed as a potential conflict of interest.

## Abbreviations

BER: base excision repair
CRISPR: clustered regularly interspaced short palindromic repeats
DDR: DNA damage response
DSB: double-strand break
ePK: eukaryotic-like protein kinase
HJ: holliday junction
HRR: homologous recombination repair
KD: protein kinase catalytic domain
MMR: mismatch repair
NHEJ: non-homology end joining
PTM: post-translational modification
Rio: right open reading frame
