# Supplementary figures and images for: Phosphorylation of the Archaeal Holliday Junction Resolvase Hjc Inhibits Its Catalytic Activity and Facilitates DNA Repair in Sulfolobus islandicus REY15A
Source: Front Microbiol. 2019 May 31;10:1214. doi: 10.3389/fmicb.2019.01214 (PMC6555300; doi:10.3389/fmicb.2019.01214)

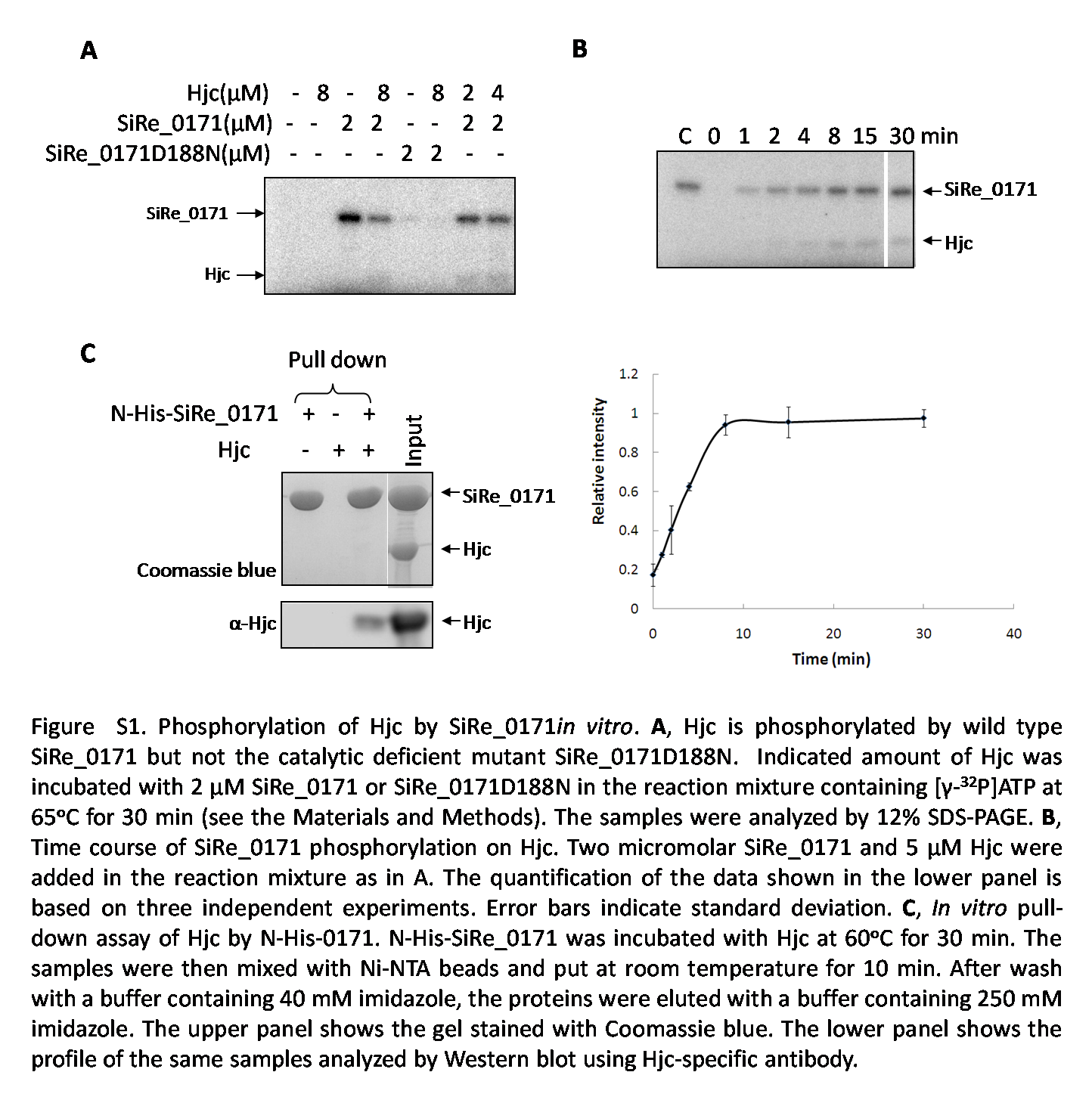

Supplement: Supplementary file 1 [file Image_1.TIF]

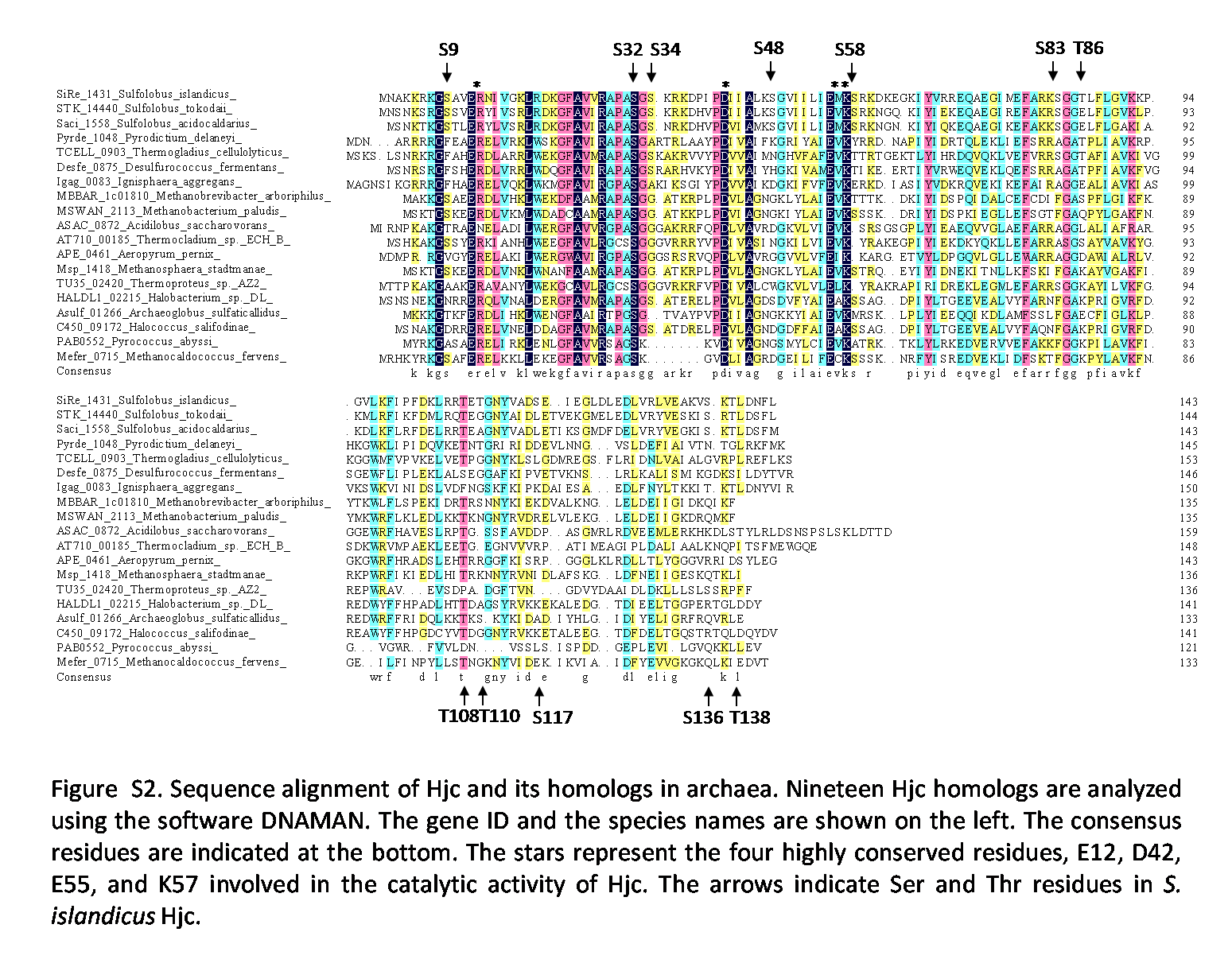

Supplement: Supplementary file 2 [file Image_2.TIF]

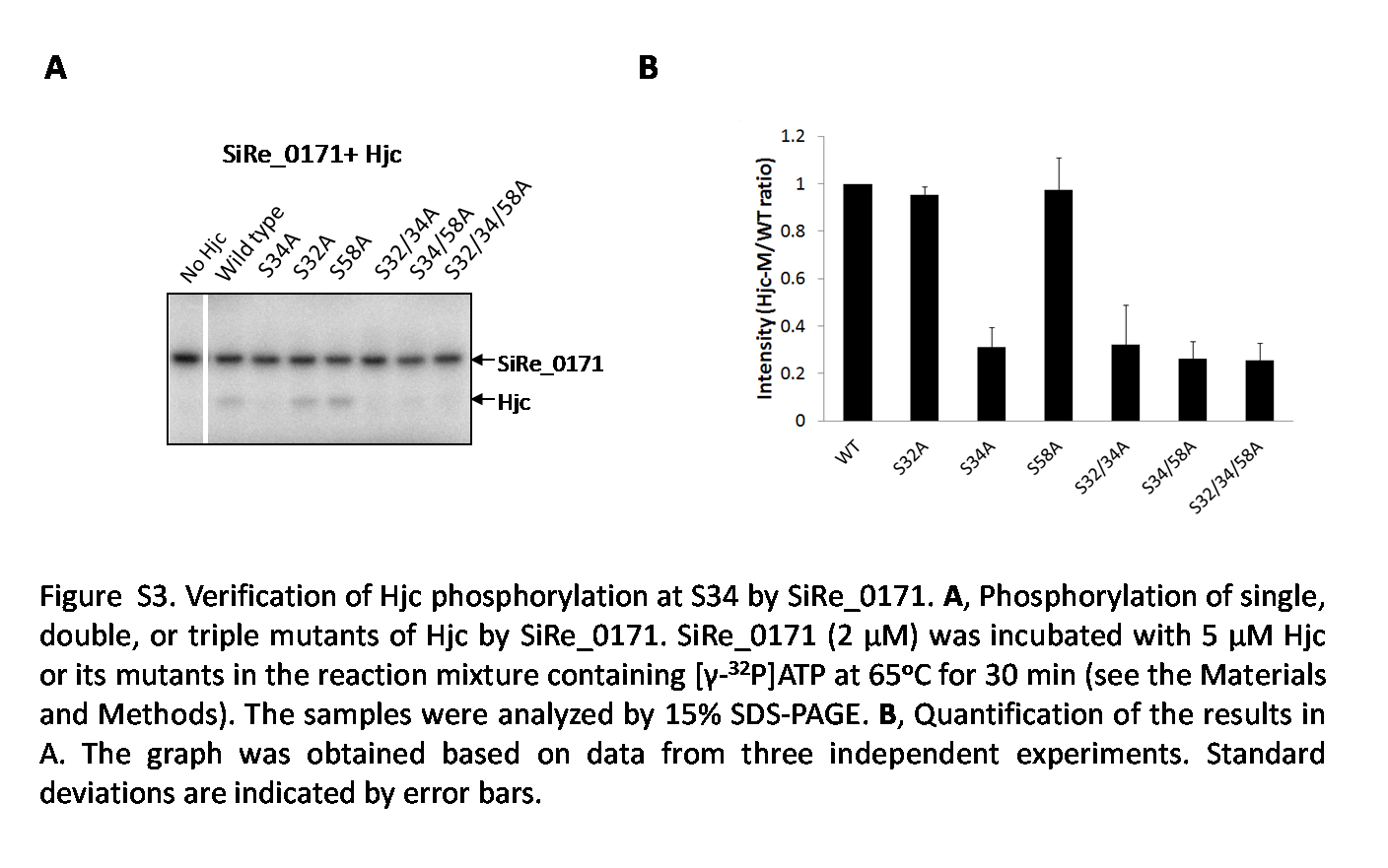

Supplement: Supplementary file 3 [file Image_3.TIF]

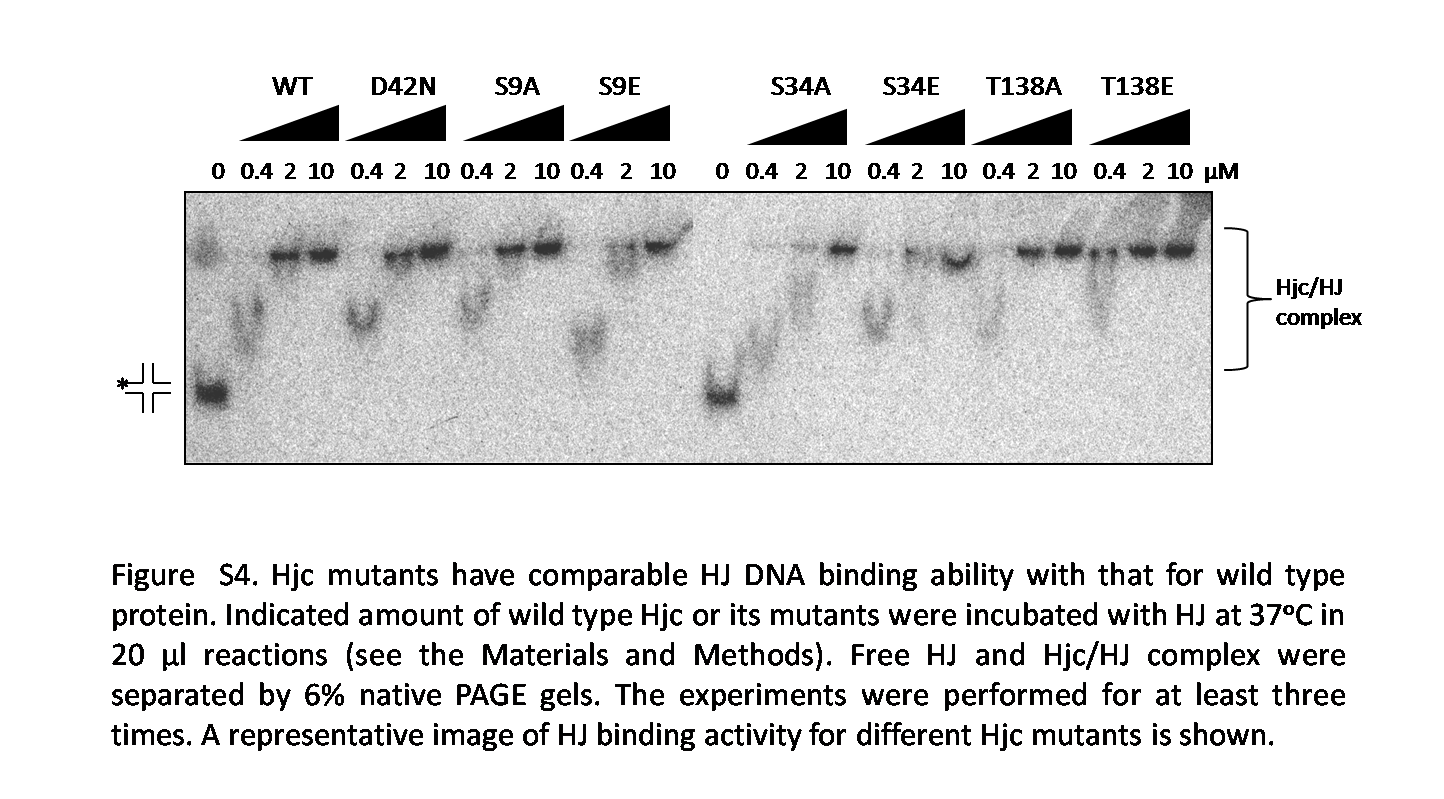

Supplement: Supplementary file 4 [file Image_4.TIF]

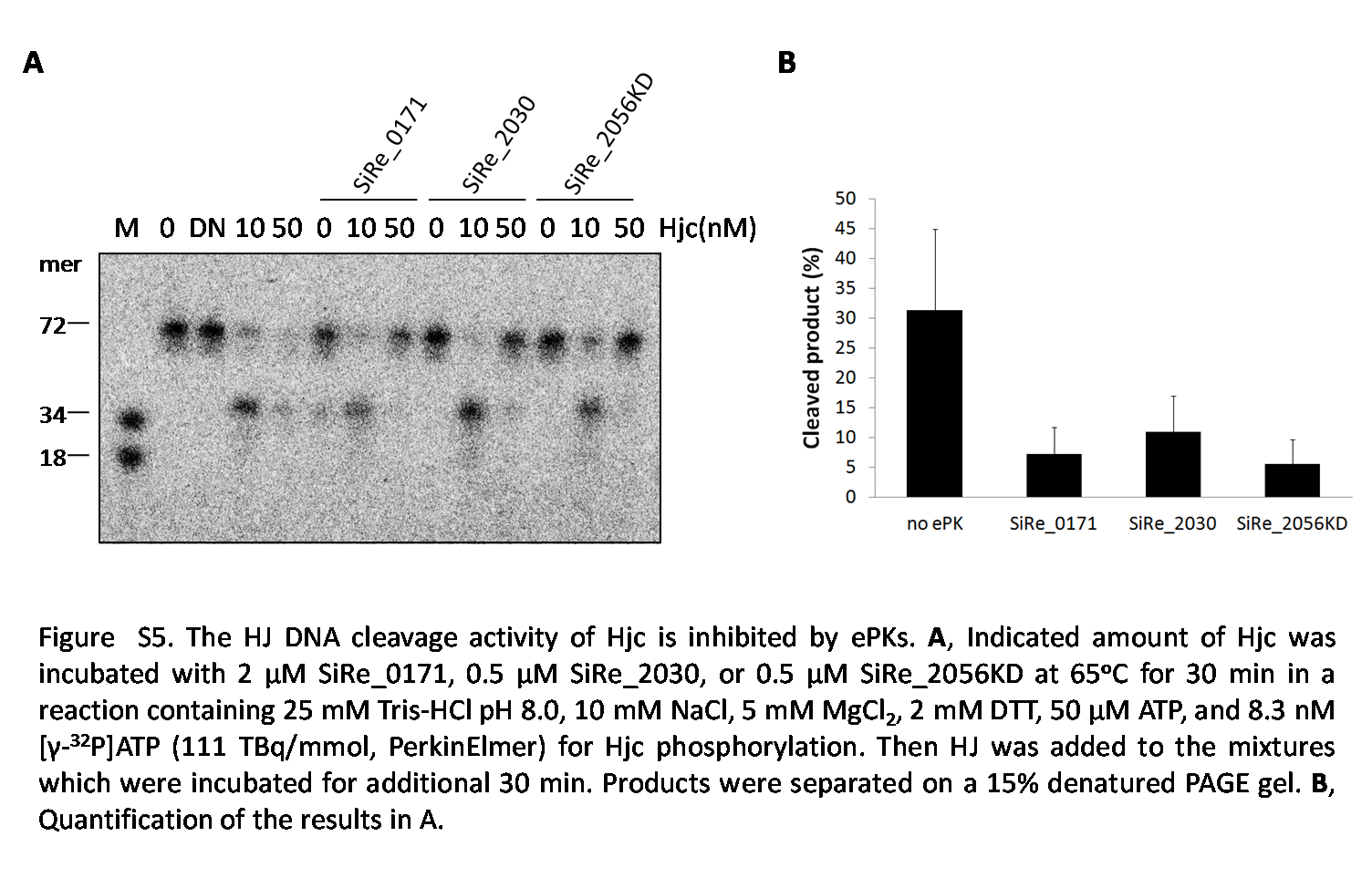

Supplement: Supplementary file 5 [file Image_5.TIF]

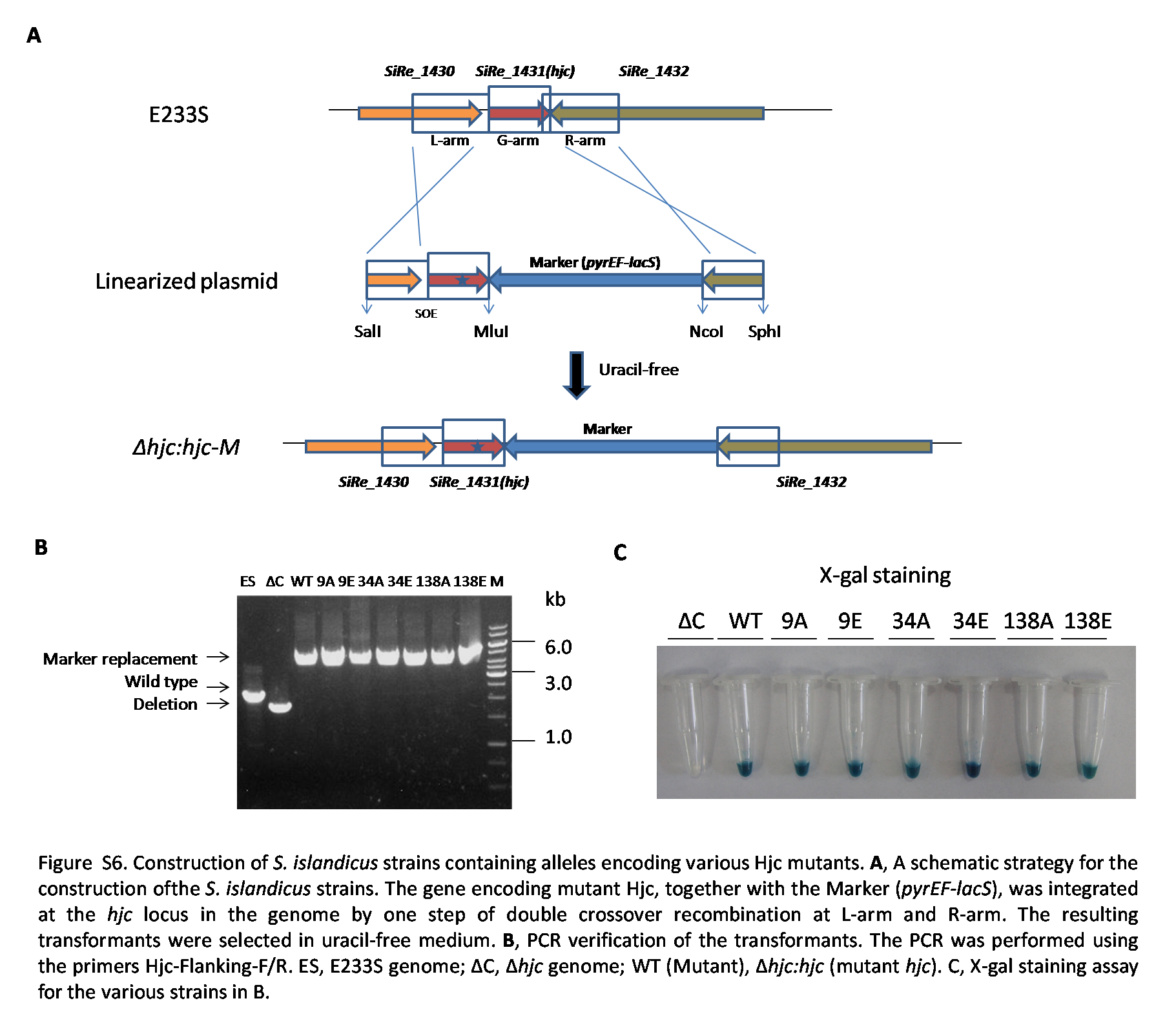

Supplement: Supplementary file 6 [file Image_6.TIF]

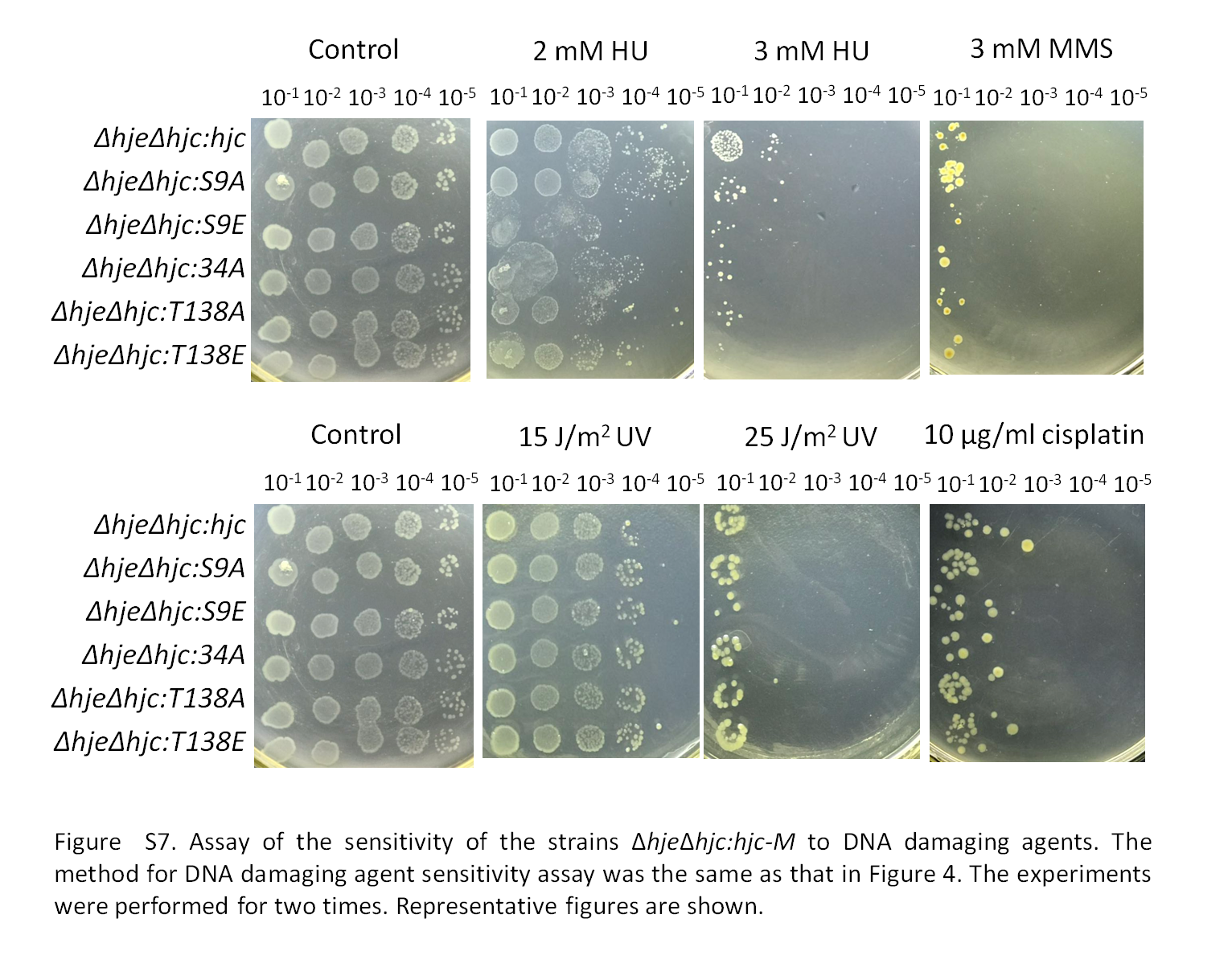

Supplement: Supplementary file 7 [file Image_7.tif]

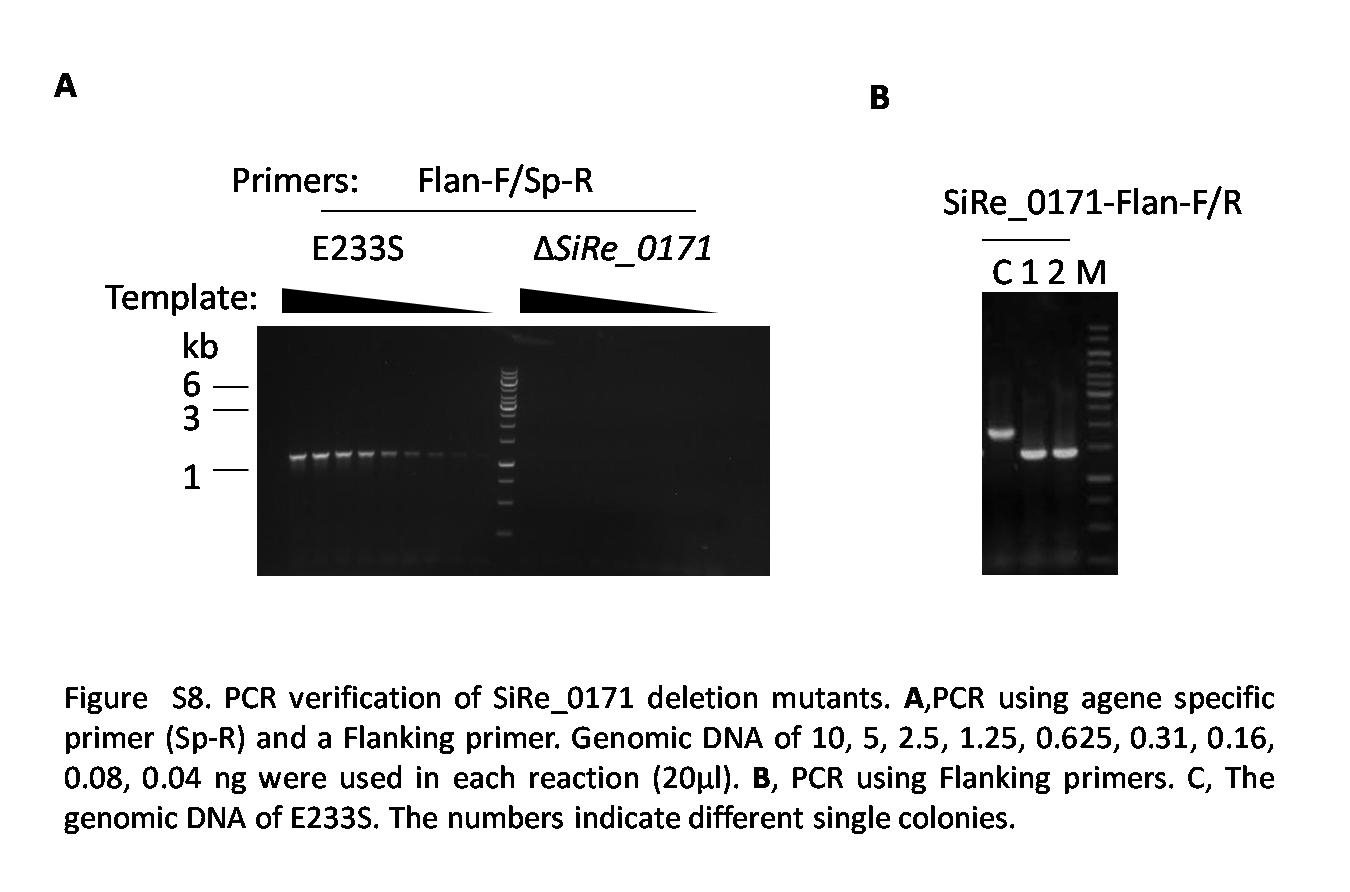

Supplement: Supplementary file 8 [file Image_8.TIF]

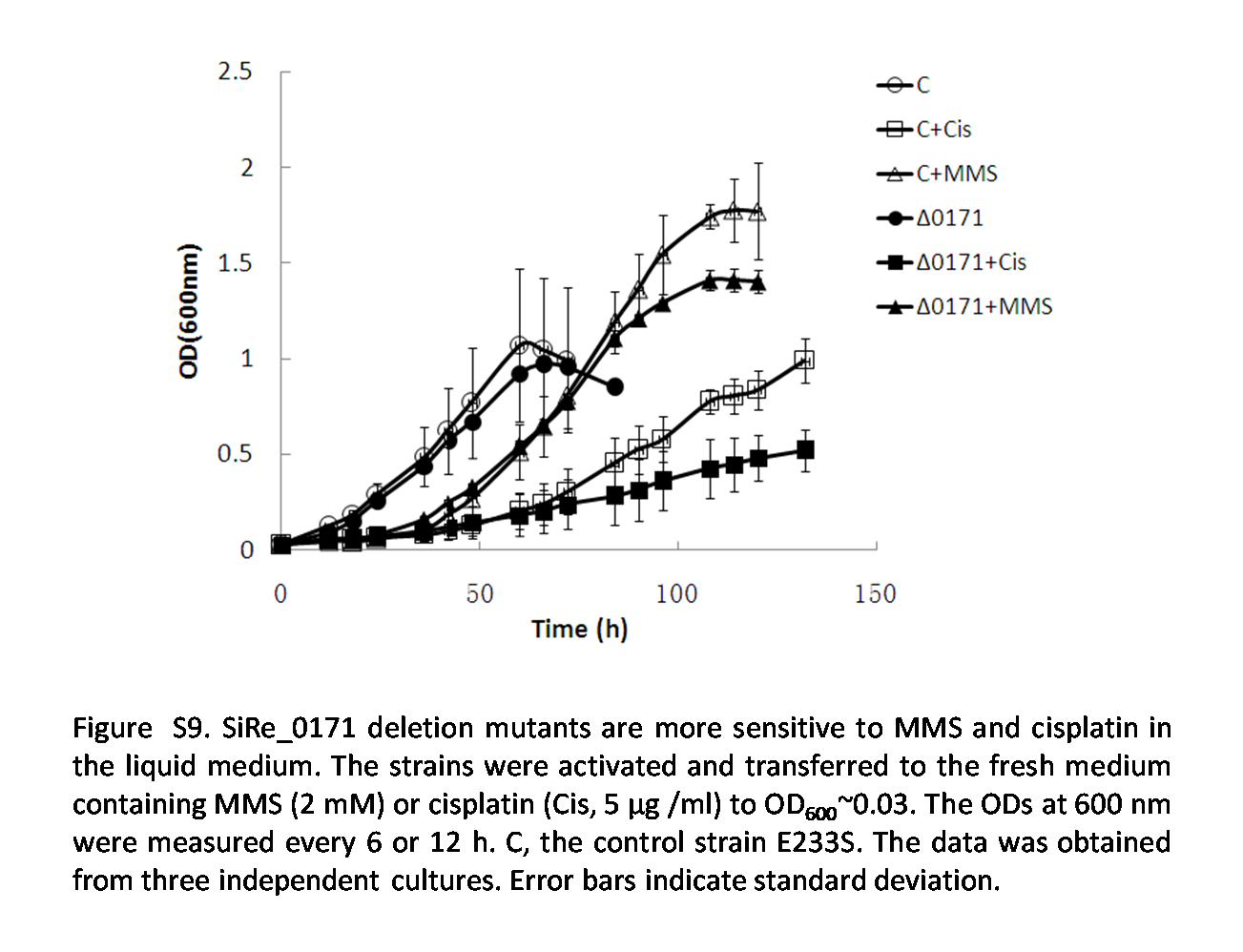

Supplement: Supplementary file 9 [file Image_9.TIF]
