# Supplementary material for: Phosphorylation of the Archaeal Holliday Junction Resolvase Hjc Inhibits Its Catalytic Activity and Facilitates DNA Repair in Sulfolobus islandicus REY15A
Source: Front Microbiol. 2019 May 31;10:1214. doi: 10.3389/fmicb.2019.01214 (PMC6555300; doi:10.3389/fmicb.2019.01214)
Supplement: Supplementary file 10 [file Table_1.DOCX]

Supplementary Material

# Supplementary Tables

**Table S1. Strains used in this study**

| **Strains** | **Genotype** | **Source or reference** |
| --- | --- | --- |
| *S. islandicus* REY15A (E233S) | Δ*pyrEF*Δ*lacS* | Deng, *et al*., 2009. |
| Δ*hjc:hjc,* Δ*hjc:S9A,* Δ*hjc:S9E,* Δ*hjc:S34A,* Δ*hjc:S34E,* Δ*hjc:T138A,* Δ*hjc:T138E* | Wild type*hjc*gene was replacedwith*hjc, S9A, S9E, S34A, S34E, T138A,* or*T138E* | This work |
| Δ*hje*Δ*hjc:hjc,* Δ*hje*Δ*hjc:S9A,* Δ*hje*Δ*hjc:S9E,* Δ*hje*Δ*hjc:S34A,* Δ*hje*Δ*hjc:S34E,* Δ*hje*Δ*hjc:T138A,* Δ*hje*Δ*hjc:T138E* | Wild type*hjc*gene inΔ*hje*was replaced with *hjc, S9A, S9E, S34A, S34E, T138A,* or*T138E* | This work |
| Δ*SiRe_0171* | *SiRe_0171* deletion mutant | This work |

**Table S2. Oligonucleotides used in this study**

| **Primer** | **Sequence ^a^ (5’-3’)** |
| --- | --- |
| SisHjc-NdeI-F | CAACGGG**CATATG**AACGCTAAAAAGAGG |
| SisHjc-SalI-R | GGTT**GTCGAC**TTAGAGAAAATTATCCAGC |
| SisHjc-D42N-R | TTTAAGGCGATAATATTCGGTATAGGGTCTT |
| SisHjc-D42N-F | AAGACCCTATACCGAATATTATCGCCTTAAA |
| SisHjc-S9A-NdeI-F | AATAAGA**CATATG**AACGCTAAAAAGAGGAAAGGTGCTGCAGTA |
| SisHjc-S9E-NdeI-F | AATAAGA**CATATG**AACGCTAAAAAGAGGAAAGGTGAGGCAGTA |
| SisHjc-S32A-R | TTTCTCTTACTTCCAGCCGCTGGTGCTCTT |
| SisHjc-S32A-F | AAGAGCACCAGCGGCTGGAAGTAAGAGAAA |
| SisHjc-S34A-R | CTTTTCTCTTAGCTCCACTCGCTGGTG |
| SisHjc-S34A-F | CACCAGCGAGTGGAGCTAAGAGAAAAG |
| SisHjc-S34E-R | CTTTTCTCTTTTCTCCACTCGCTGGTG |
| SisHjc-S34E-F | CACCAGCGAGTGGAGAAAAGAGAAAAG |
| SisHjc-S48A-R | TAGGATAATAACACCAGCCTTTAAGGCGAT |
| SisHjc-S48A-F | ATCGCCTTAAAGGCTGGTGTTATTATCCTA |
| SisHjc-S58A-R | TCTTTATCTTTTCTAGCCTTCATTTCAATTAGG |
| SisHjc-S58A-F | CCTAATTGAAATGAAGGCTAGAAAAGATAAAGA |
| SisHjc-S58E-R | TCTTTATCTTTTCTTTCCTTCATTTCAATTAGG |
| SisHjc-S58E-F | CCTAATTGAAATGAAGGAAAGAAAAGATAAAGA |
| SisHjc-S32AS34A-R | CTTTTCTCTTAGCTCCAGCCGCTGGTGCT |
| SisHjc-S32AS34A-F | AGCACCAGCGGCTGGAGCTAAGAGAAAAG |
| SisHjc-S83AS86A-R | CTAAAAATAAAGCCCCACCAGCTTTTCTTGC |
| SisHjc-S83AS86A-F | GCAAGAAAAGCTGGTGGGGCTTTATTTTTAG |
| SisHjc-T108A-R | TAATTCCCAGTTTCAGCCCTTCTTAACTTATC |
| SisHjc-T108A-F | GATAAGTTAAGAAGGGCTGAAACTGGGAATTA |
| SisHjc-T108AT110A-R | ATAATTCCCAGCTTCAGCCCTTCTTAACTTA |
| SisHjc-T108AT110A-F | TAAGTTAAGAAGGGCTGAAGCTGGGAATTAT |
| SisHjc-S117A-R | AACCCTTCTATCTCAGCGTCTGCAACATAA |
| SisHjc-S117A-F | TTATGTTGCAGACGCTGAGATAGAAGGGTT |
| SisHjc-S136A-R | TATT**GTCGAC**TTAGAGAAAATTATCCAGCGTTTTGGCCACTTT |
| SisHjc-T138A-R | TATT**GTCGAC**TTAGAGAAAATTATCCAGAGCTTTGCTCACTTT |
| SisHjc-T138E-R | TATT**GTCGAC**TTAGAGAAAATTATCCAGTTCTTTGCTCACTTT |
| SisHjc-S136AT138A-R | TATT**GTCGAC**TTAGAGAAAATTATCCAGAGCTTTGGCCACTTT |
| SisHjc-L-arm-SalI-F | ATTG**GTCGAC**GTGGTACAGCTCTTCAAAAG |
| SisHjc-L-G-SOE-R | CCTCTTTTTAGCGTTCATAAGGTATTATATAAAT |
| SisHjc-L-G-SOE-F | ATTTATATAATACCTTATGAACGCTAAAAAGAGG |
| SisHjc-G-arm-MluI-R | TTGC**ACGCGT**TTAGAGAAAATTATCCAG |
| SisHjc-R-arm-NcoI-F | TTGC**CCATGG**CGCTGGATAATTTTCTCTA |
| SisHjc-R-arm-SphI-R | TGAT**GCATGC**GAGATGAGAAATGACGAGG |
| SisHjc-Flanking-F | GGGAATTGGCTAAGAGCGATGGGAT |
| SisHjc-Flanking-R | GACCAGTAGTTAGGAAAAGATTAGAGGA |
| SiRe_0171-protospacer-F | AAAGaaacctattctggtttacgaaaacattcttgtaatggaat |
| SiRe_0171-protospacer-R | TAGCATTCCATTACAAGAATGTTTTCGTAAACCAGAATAGGTTT |
| SiRe_0171-L-arm-SalI-F | TTTA**GTCGAC**ATGGAATTGGAGGATCAACT |
| SiRe_0171-R-arm-NotI-R | TATTTATA**GCGGCCGC**AGACTGAGGAATGT |
| SiRe_0171-L-R-arm-SOE-F | ATTCAAATAGCTAGAAGCTCGAGGATGTGAGCCAAGCAATT |
| SiRe_0171-L-R-arm-SOE-R | AATTGCTTGGCTCACATCCTCGAGCTTCTAGCTATTTGAAT |
| SiRe_0171-Flanking-F | GATAGACTTAACGTTTTTGGAGGGGC |
| SiRe_0171-Flanking-R | CACTTACCGATTGCTCTTGATCTCGT |
| Strand 5 (72-mer) | CGAGCGACAGGAACCTCGAGAAGCTTCAATCGGCTCAGACCGAGCAGAATTCTATGTGTTTACCAAGCGCTG |

| Strand 6 (72-mer) | CAGCGCTTGGTAAACACATAGAATTCTGCTCGGTCTCTCGGCAGATTCTAGAAATCGACGCTAGCAAGTGAC |
| --- | --- |

| Strand 7 (72-mer) | GTCACTTGCTAGCGTCGATTTCTAGAATCTGCCGAGACTGGCTGTGGGATCCGAGCTGTCTAGAGACATCGA |
| --- | --- |
| Strand 8 (72-mer) | TCGATGTCTCTAGACAGCTCGGATCCCACAGCCAGTGAGCCGATTGAAGCTTCTCGAGGTTCCTGTCGCTCG |
| 34-mer marker | CAAGCTTGCATGCCTGCAGGTCGACTCTAGAGGA |

^a^Restriction sites are indicated in boldface and mutated codons for construction of site-directed mutantsare underlined.
